# Zinc Finger 280B Regulates sGCα1 and p53 in Prostate Cancer Cells

**DOI:** 10.1371/journal.pone.0078766

**Published:** 2013-11-13

**Authors:** Shuai Gao, Chen-Lin Hsieh, Jun Zhou, Lirim Shemshedini

**Affiliations:** Department of Biological Sciences, University of Toledo, Toledo, Ohio, United States of America; Innsbruck Medical University, Austria

## Abstract

The Zinc Finger (ZNF) 280B protein was identified as an unexpected target of an shRNA designed for sGCα1. Further analysis showed that these two proteins are connected in another way, with 280B up-regulation of sGCα1 expression. Knock-down and over-expression experiments showed that 280B serves pro-growth and pro-survival functions in prostate cancer. Surprisingly however, these pro-cancer functions of 280B are not mediated by sGCα1, which itself has similar functions in prostate cancer, but by down-regulated p53. The p53 protein is a second target of 280B in prostate cancer, but unlike sGCα1, p53 is down-regulated by 280B. 280B induces p53 nuclear export, leading to subsequent proteasomal degradation. The protein responsible for p53 regulation by 280B is Mdm2, the E3 ubiquitin ligase that promotes p53 degradation by inducing its nuclear export. We show here that 280B up-regulates expression of Mdm2 in prostate cancer cells, and this regulation is via the Mdm2 promoter. To demonstrate an *in vivo* relevance to this interaction, expression studies show that 280B protein levels are up-regulated in prostate cancer and these levels correspond to reduced levels of p53. Thus, by enhancing the expression of Mdm2, the uncharacterized 280B protein provides a novel mechanism of p53 suppression in prostate cancer.

## Introduction

The p53 tumor suppressor has a pivotal role in coordinating cellular responses to various stresses (including DNA damage, over-expressed oncogenes and diverse metabolic limitations) [1]. In response to cellular stresses, p53 expression is induced and the protein is translocated into the nucleus to bind to DNA in a sequence-specific manner, thereby activating or repressing target genes [2]. Thus, p53 can orchestrate biological outputs such as apoptosis, cell-cycle arrest, senescence, or modulation of autophagy. Several downstream direct targets are involved in the anti-proliferative function of p53, including pro-survival *suvivin* [3] and the cell cycle inhibitor *p21/CIP1* [4].

Mdm2 (Mouse double minute 2 homolog) is the major regulator of p53 [5]. Principally, Mdm2 is an E3 ligase that down-regulates p53 through nuclear export and ubiquitin-dependent proteasomal degradation [6]–[9]. In addition, the histone acetyl transferase (HAT) proteins p300 and CBP (CREB-binding protein) stabilize p53 through acetylation, and this stabilization effect can be abrogated by Mdm2 through formation of a ternary complex with p53 and p300 or CBP [10], [11]. Mutations in p53 that that lead to loss-of-function represent the most common genetic change in human cancers [12], [13]. Interestingly, a complete loss of p53 is found only in advanced prostate cancer [14]. It was later determined that a combined loss of p53 and PTEN, a tumor suppressor involved in PI3-AKT signaling [15], was sufficient to drive development of invasive prostate cancer [16]. Another tumor suppressor, NKX3.1, was shown to increase acetylation and stability of p53 via Mdm2-dependent mechanisms [17].

Soluble guanylyl cyclase (sGC) is the nitric oxide (NO) receptor, and is well known for its enzymatic activity to convert guanosine 5′-triphosphate (GTP) to cyclic guanosine 3′,5′-monophosphate (cGMP). This pathway is mostly involved in the cardiovascular and nervous systems [18]. We have previously reported that the α1 subunit of SGC (sGCα1; gene name *GUCY1A3*) is a direct target of androgen receptor (AR) and plays important role in driving prostate cancer cell proliferation [19]. We also determined that sGCα1 serves a pro-survival function in prostate cancer by inhibiting p53 activity and selectively suppressing genes that are involved in apoptosis, via a mechanism of p53 cytoplasmic sequestration [20]. Interestingly, these pro-cancer functions of sGCα1 are independent of sGC enzyme activity. More recently, the therapeutic potential of targeting sGCα1 was developed via a binding peptide that has strong anti-cancer activity [21].

Zinc Fingers (ZNF) are a common DNA-binding motif found in several transcription factors, and the C_2_H_2_ motif is the most common type [22]. This motif is characterized by two cysteine and two histidine residues coordinating one or more zinc ions and projecting into a finger-like structure that interacts with DNA [23]. Evidence is emerging that ZNF proteins play important roles in human cancers. For example, ZNF23 inhibits tumor cell growth through enhancement of p27/kip-1 expression, and the expression levels of ZNF23 protein are greatly reduced in human cancer [24]. In colorectal cancer, ZKSCAN3 (ZNF306) expression was elevated and under-expression of ZKSCAN3 reduced tumorigenicity [25]. Furthermore, several reports show that ZNF proteins can affect p53 activity. ZNF668 was identified as a tumor suppressor in breast cancer, which stabilizes p53 by preventing Mdm2-mediated p53 ubiquitination and degradation [26]. ZNF307 suppresses the activity of p53 and p21 by increasing transcription of Mdm2 and EP300 [22].

While studying the function of sGCα1 using RNAi, we identified Zinc Finger 280B (ZNF280B, thereafter called 280B) as a target of one of the sGCα1 shRNAs. The limited information available on 280B and the possible relationship of ZNF proteins with p53 encouraged us to further analyze 280B for a potential role in prostate cancer. In this study, we show a functional interaction among 280B, p53, and Mdm2. Over-expression of 280B significantly decreased p53 levels by decreasing its stability. This effect on p53 stability is mediated by the ability of 280B to induce the expression of Mdm2, through a positive effect on the Mdm2 promoter. By suppressing p53, 280B provides pro-survival and pro-growth functions in prostate cancer. In support of this, high expression of 280B is associated with low p53 levels in prostate tumor tissues.

## Materials and Methods

### Cell culture and siRNA transfection

LNCaP, C81 and CWR-22Rv1 cells were grown as previously described [20]. Normal prostate epithelial cells (PrEC) were cultured in PrEGM as recommended by supplier (Lonza). Scrambled Control siRNA, sGCα1 siRNA, MDM2 siRNA (GAACAAGAGACCCUGGUUA) and ZNF280B siRNA (GGAACAAUCAUGUGAGGAA) (Dharmacon) at 40 nM final concentration was transfected into cells using Lipofectamine siMAX (Invitrogen).

### Reporter Assay and Plasmid Transfection

C81 cells were grown to 80–90% confluence in RPMI-1640 with 5% FBS. After 24 hrs, medium was replaced with serum-free medium and the cells were transiently transfected with the p53-Luc reporter plasmid or Mdm2-Luc reporter plasmid, and control siRNA, or siRNA against ZNF280B and p53. The pCH110 plasmid encoding b-galactosidase was used to monitor transfection efficiency [27]. The p53-Luc plasmid was kindly provided by Dr. Andrei Gudkov and contains three p53-responsive elements. The Mdm2-Luc and Mdm2 expression plasmid was kindly provided by Dr. Jason M. Shohet. The transfection was performed by using Lipofectamine 2000 and luciferase activity was measured using luciferase assay system from Promega.

### Adenovirus and Lentivirus Infections

C81 cells were infected with 5–50 MOI of either an adenovirus expressing ZNF280B (ABMGood)) or an empty adenovirus as a control. After 48 hours, the cells were subjected to RT-PCR and western blotting.

sGC**α**1 shRNA, empty shRNA, and package vectors were purchased from Open Biosystems. The lentiviral particles were prepared following the Company's protocol. LNCaP cells were infected with lentivirus and selected using puromycin at a concentration of 4 mg/ml. After 4 days of selection, the cells were subjected to MTT assay, qRT-PCR, Western blotting.

### Immunohistochemistry

Immunohistochemistry was used to compare the level of ZNF280B in normal prostate tissues and tumor tissues obtained from the CHTN. ZNF280B antibody (1∶200 dilution; Sigma) was used for immunohistochemisty as previously described [27].

### Proliferation assays

After siRNA transfection for 3 days, 20,000 cells from each condition were seeded in 24-well plates. The MTT assay (Sigma) was used as before [21] to determine cell number.

### Western blotting and Cell Fractionation

Western blotting was performed as described [19] using primary antibodies against sGCα1 (Cayman Chemical), ZNF280B (Abgent), MDM2 (Santa Cruz), Survivin (Cell Signaling Technology), p21 (Cell Signaling Technology), p53 (Santa Cruz), β-Tubulin (Abcam), RARα (Santa Cruz), β-Actin (Abcam).

C81 cells treated with siRNA and adenovirus were harvested and washed once with cold PBS. 10% of the cells were saved as input and the remaining portion was divided into nuclear and cytosolic fractions using Nuclear/Cytotsol Fractionation Kit (MBL International). The fractions were then subjected to Western Blotting to measure ZNF280B and p53 protein levels.

### Semi-Quantitative RT-PCR and QRT-PCR

Total mRNA was isolated using Trizol reagent following protocol from manufacture (Invitrogen), and subjected to semi-quantitative RT-PCR and QRT- PCR analyses as described [28]. The PCR upstream and downstream primers used for each gene were: *Survivin*, 5′-GGACCACCGCATCTCTACAT-3′ and 5′-GACAGAAAGGAAAGCGCAAC-3′; *p53*, 5′-GGCCCACT TCACCGTACTAA-3′ and 5′-G TGGTTTCAAGGCCAGATGT-3′; *sGCα 1*, 5′-AGCAGTGTGGAGAGCTGGAT-3; and 5′-CTGATCCAGAGTGCAGTCCA-3′; *p21*, 5′-GACACCACTGGAGGGTG ACT-3′ and 5′-CAGGTCCACATGGTCTTCCT; *GAPDH*, 5′-CGACCACTT TGTCAAGCTCA-3′ and 5′-AGGGGAGATTCAGTGTGGTG-3′. *MDM2*, 5′-TTTCGCAGCCAGGAGCACCG-3′ and 5′-AGTTTCCTTCACGGGGCGCG-3′; *ZNF280B*, 5′-TCCCAAAAGGGCTAAACTCA-3′ and 5′GTCCTTTGGAAAAGC- TGCTG-3′.

## Results

### An sGCα1 siRNA targets 280B in prostate cancer cells

**Figure 1:**
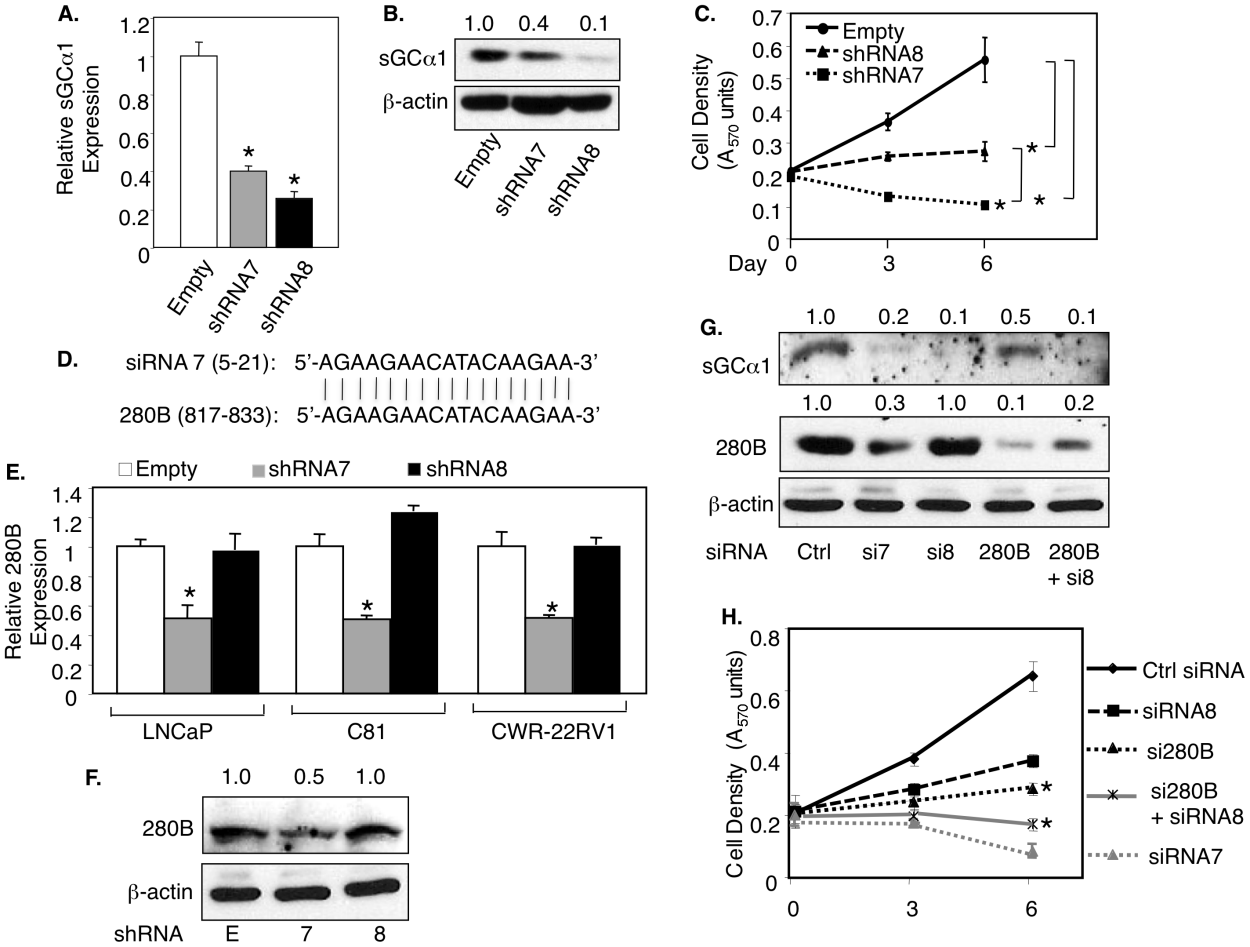
An sGCα1 siRNA also targets 280B in prostate cancer cells. (**A, B, C**) LNCaP cells were infected with empty lentivirus or lentiviruses expressing one of two different sGCα1 shRNAs (7, 8) and sGCα1 expression was measured by real-time PCR (A) or Western blotting (B), and cell density was measured by MTT assay (C). (**D**) The diagram shows the nucleotide homology between siRNA 7 and 280B. (**E, F**) LNCaP, C81, and CWR22-RV1 cells were subjected to the same infections and 280B levels were measured by real-time PCR (E) or Western blotting (F). (**G, H**) C81 cells were transfected with Control (Ctrl), sGCα1 siRNA (7,8), 280B siRNA, and 280B plus sGCα1 siRNA 8, and sGCα1 levels were measured by Western blotting (G), and cell density was measure by MTT assay (H). All expression levels are relative to the first condition (A, D), and it was set to 1. β-actin served as a loading control in the Western blots. Bar graphs represent averages of three independent experiments plus SD. Asterisks indicate statistical significance (P<0.01).

To study the role of endogenous sGCα1 in prostate cancer cells, we used shRNA to knockdown this gene in LNCaP cells. Among five different shRNAs, two were most effective in down-regulating sGCα1 protein expression: shRNA7 and shRNA8 (data not shown). These shRNAs were used for further study. As shown in Fig. 1, shRNA8 was more effective than shRNA7 at diminishing both the levels of sGCα1 mRNA (Fig. 1A) and protein (Fig. 1B); similar results were observed with siRNA7 and siRNA8 (Fig. S1B, C). Interestingly, however, shRNA7 (and siRNA7) was significantly stronger than shRNA8 (and siRNA8) at blocking the proliferation of LNCaP cells (Fig. 1C and Fig. S1C). This surprising result led us to consider the possibility of off-target effects of either or both shRNAs. A Blast search identified that 17 of 21 nucleotides of siRNA7 matched perfectly with part of the 280B gene (Fig. 1D); a significant part of siRNA8 matched the HIF1A gene (data not shown). shRNA7 and siRNA7 were able to significantly down-regulate 280B both at the level of mRNA (Fig. 1E) and protein (Fig. 1F), while shRNA8/siRNA8 had no effect, in LNCaP cells and the two hormone-independent cell lines C81 and CWR-22Rv1; interestingly, neither shRNA8 nor shRNA7 affected HIF1A expression (data not shown). In view of these data, we hypothesized that the higher negative effect on cell growth of shRNA7/siRNA7, as compared to shRNA8/siRNA8, is due to the added effect of shRNA7/siRNA7 on 280B. To test this hypothesis, we used a 280B-specific siRNA, which interestingly has a significant negative effect on LNCaP cell proliferation (Fig. 1H). The hypothesis was directly tested by performing a double knockdown of sGCα1 using the siRNA8 and the 280B-specific siRNA (Fig. 1G), which blocked cell proliferation more strongly than either siRNA8 or the 280B siRNA alone and nearly to the same extent as siRNA7 (Fig. 1H). These data strongly suggest that 280B serves a pro-growth function in prostate cancer cells.

### 280B up-regulates sGCα1 mRNA and protein in prostate cancer cells

**Figure 2:**
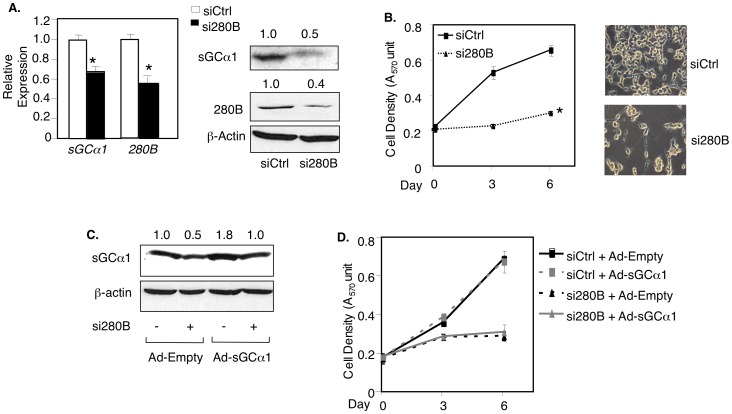
280B up-regulates sGCα1 in prostate cancer cells. (**A, B**) LNCaP cells were transfected with control or 280B siRNA and sGCα1 levels were measured by real-time PCR or Western blotting (A) and cell density was measured by MTT assay (B); phase contrast pictures were taken on day 6 (B). (**C, D**) LNCaP cells were infected with Empty or sGCα1 adenovirus after transfection with control (−) or 280B siRNA (+), and sGCα1 levels were measured by Western blotting (C), and cell density was measure by MTT assay (D). All expression levels are relative to the first condition (A), and it was set to 1. β-actin served as a loading control in the Western blots. Bar graphs represent averages of three independent experiments plus SD. Asterisks indicate statistical significance (P<0.01).

To study the role of 280B in prostate cancer cells, we used the 280B-specific siRNA (from Fig. 1G). The 280B siRNA was able to down-regulate, as expected, 280B mRNA and protein (Fig. 2A) and, surprisingly, sGCα1 expression (Fig. 2A). A Blast search of the 280B siRNA sequence identified only the 280B gene as a direct target and clearly showed that sGCα1 does not have any sequence similarity to the 280B siRNA sequence. These data suggest that the sGCα1 mRNA and protein are a target of the 280B protein, not the siRNA.

To determine if the down-regulated sGCα1 is responsible for the reduced cell proliferation resulting from 280B knockdown, we repeated the transfection with the 280B siRNA, which as before (see Fig. 1H) resulted in a marked slow-down in LNCaP cell growth (Fig. 2B). Indeed, the cells treated with 280B siRNA changed their morphology and began to die (Fig. 2B). To determine if sGCα1 over-expression can rescue the cells, siRNA-transfected cells were infected with sGCα1 adenovirus, resulting in recovered sGCα1 protein levels (Fig. 2C). Over-expression of sGCα1 failed to rescue the cells (Fig. 2D), indicating that sGCα1 is either not able or not sufficient to rescue prostate cancer cells having reduced 280B expression and thus suggest that another 280B target is involved.

### 280B down-regulates p53 protein in prostate cancer cells

**Figure 3:**
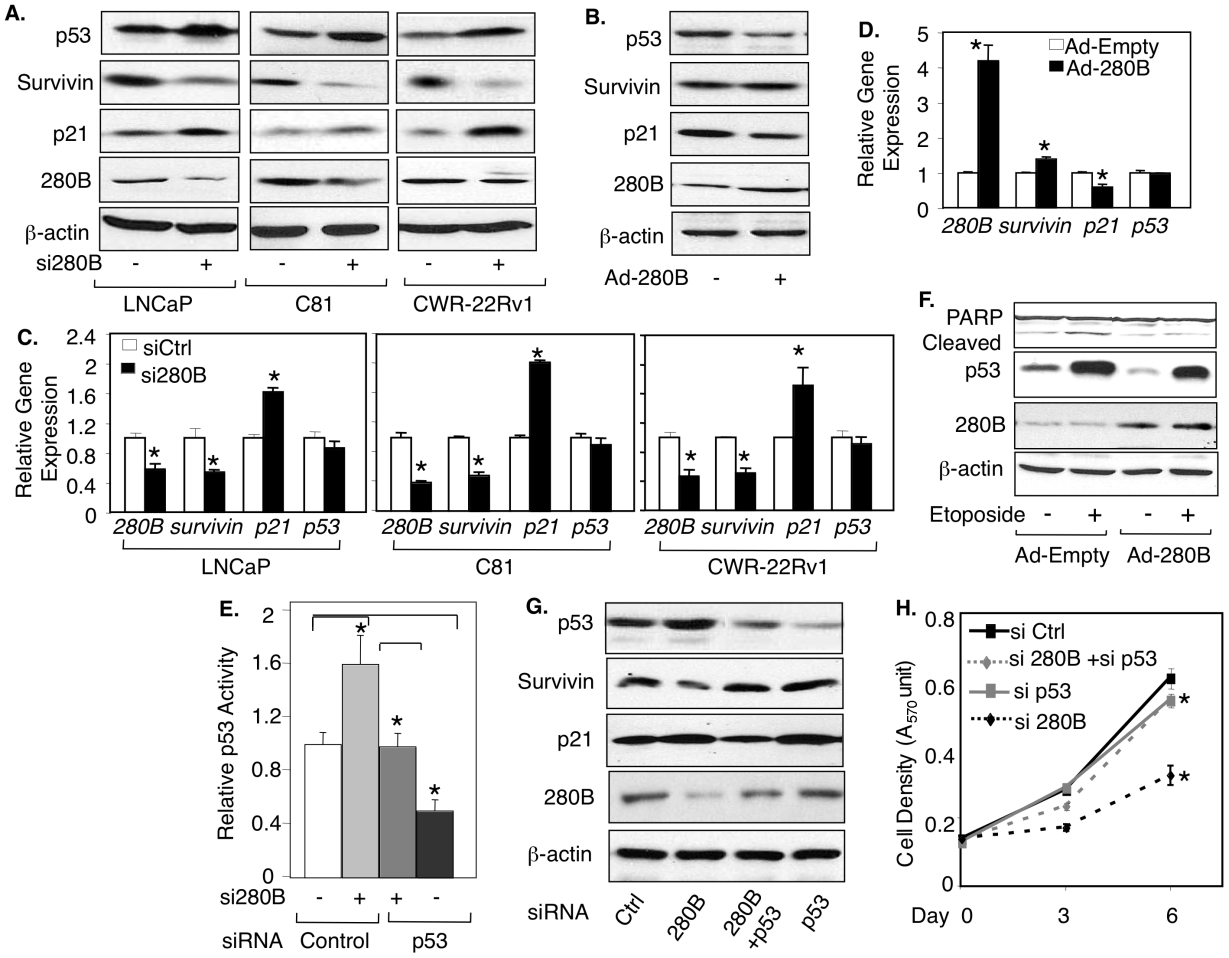
280B down-regulates p53 signaling in prostate cancer cells. (**A, C**) LNCaP, C81, CWR22-RV1 cells were transfected with control (−) or 280B siRNA (+) and p53, survivin, p21, and 280B expression levels were measured by Western blotting (A) or real-time PCR (C). (**B, D**) C81 cells were infected with empty (−) or 280B adenovirus (+) and p53, survivin, p21 and 280B expression levels were measured by Western blotting (B) or real-time PCR (D). All expression levels are relative to the first condition (C and D), and it was set to 1. (**E**) C81 cells were transfected with 0.2 µg p53-Luc and control (−), or 280B (+) siRNA, in the presence of control or p53 siRNA. Transcriptional activity of p53 activity was quantified by measuring Luciferase activity. All activities are relative to the first condition, and this activity was set to 1. (**F, G**) Cells from (E) were subjected to Western blotting for p53, Survivin, p21, and 280B (F), and measurement of cell density by MTT assay (G). β-actin served as a loading control in the Western blots. Bar graphs represent averages of three independent experiments plus SD. Asterisks indicate statistical significance (P<0.02).

Since our previous work showed that sGCα1 inhibits p53 activity in prostate cancer [20] and other work has indicated that ZNF proteins can affect the p53 signaling pathway [29], we wanted to determine if 280B affects p53. Interestingly, siRNA knock-down of 280B led to increased p53 protein levels in LNCaP, C81 and CWR-22RV1 cells (Fig. 3A). By contrast, adenovirus-mediated over-expression of 280B markedly reduced p53 protein levels (Fig. 3B). The effect was only observed on the p53 protein, since p53 mRNA levels were not affected by 280B (Fig. 3C, D). Collectively, these data show that 280B negatively regulates p53 only at the protein level and is thus distinct from its effect on sGCα1, which is positive and observed on both the mRNA and protein levels.

Since 280B alters p53 protein levels, we would expect it to affect p53 transcriptional activity. This was monitored by reporter gene assay or expression of endogenous p53-regulated genes. Using a luciferase reporter plasmid containing p53-responsive elements [30], we observed a significant increase in p53 activity in response to 280B siRNA transfection (Fig. 3E). Transient transfection of 280B expression plasmid caused a small, but significant decrease in p53 activity (Fig. S2). To study endogenous genes, we focused on the expression of survivin and p21, since 280B siRNA significantly inhibited cell growth (see Fig. 2B). Survivin is a p53-repressed gene that mediates cell survival and p21 is a p53-induced gene that inhibits cell cycle progression. In response to 280B depletion, Survivin protein was reduced and p21 was increased in all three cell lines (Fig. 3A). Real-time PCR data confirmed that 280B affects survivin and p21 at the mRNA level (Fig. 3C). Meanwhile, the opposite effects were observed when the cells were treated with 280B over-expression, yielding increased Survivin and reduced p21 proteins (Fig. 3B) and mRNAs (Fig. 3D).

These findings suggest that 280B may serve a pro-survival function in prostate cancer. To test this hypothesis, we monitored apoptosis by measuring PARP cleavage. Both LNCaP and CWR-22Rv1 cells exhibited elevated levels of cleaved PARP in response to 280B knockdown (Fig. S3). To study the role of p53 in this process, we used Etoposide, which as expected, induced high levels of p53 and elevated PARP cleavage (Fig. 3F). Importantly, these effects were diminished or gone when 280B was over-expressed (Fig. 3F), clearly demonstrating that 280B can protect cells from Etoposide-induced apoptosis.

We next explored if p53 is involved in the growth inhibition induced by the 280B siRNA. To do this, we used siRNA to diminish expression of endogenous p53 in C81 cells, which was confirmed by reduced reporter gene activity (Fig. 3E) and the expression of p53-regulated proteins (Fig. 3G). Significantly, siRNA knockdown of p53 was able to almost completely rescue cell growth inhibited by 280B siRNA depletion (Fig. 3H), strongly suggesting that elevated p53 is responsible for the cell growth suppressed by 280B knockdown.

### 280B destabilizes the p53 protein in prostate cancer cells by up-regulating Mdm2

**Figure 4:**
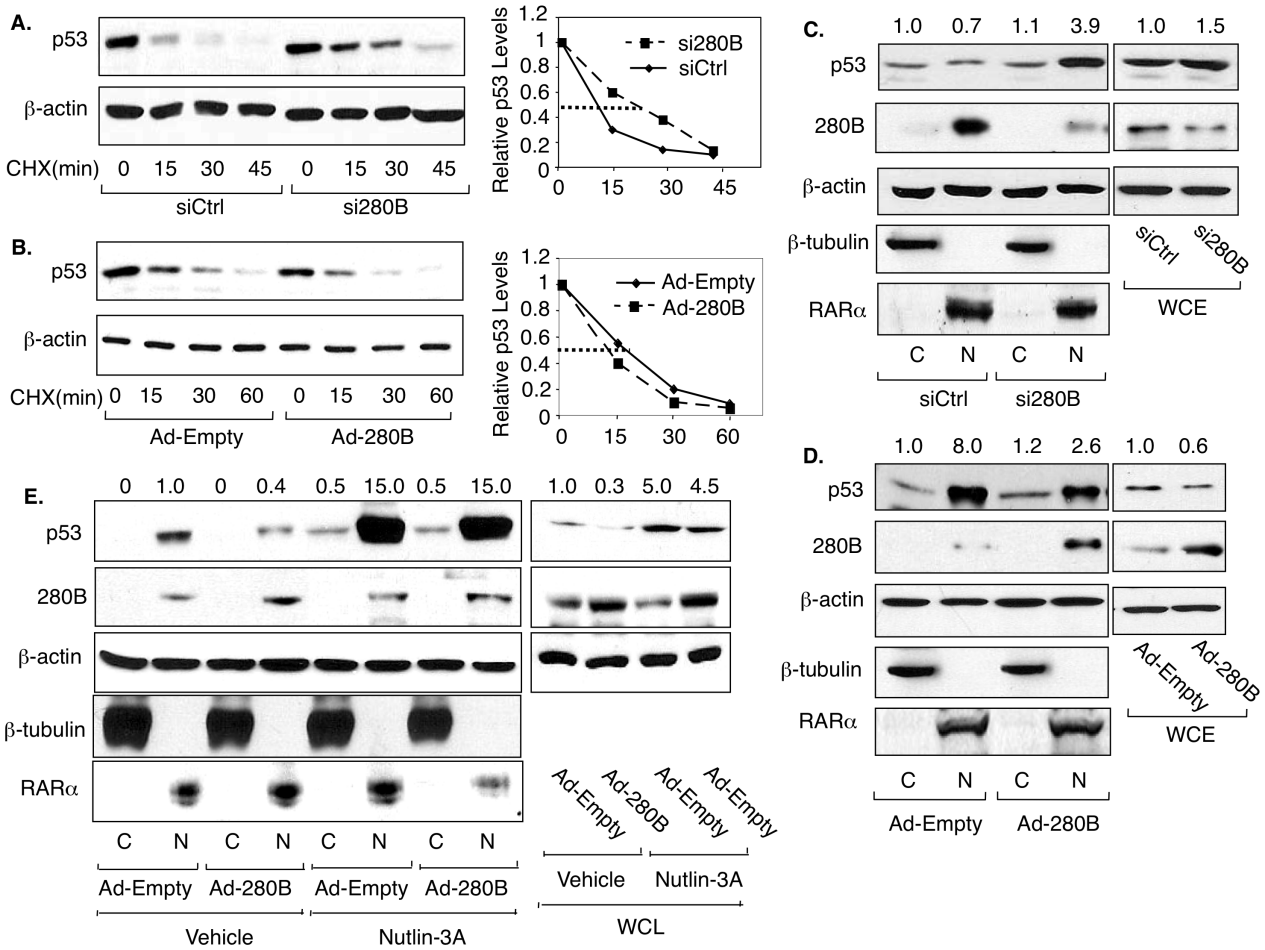
280B destabilizes the p53 protein in prostate cancer cells. (**A, B**) C81 cells were transfected with control siRNA or 280B siRNA (A) or infected with empty adenovirus or 280B adenovirus (B) for 48 hours and then treated with 100 mg/ml cycloheximide (CHX) for the indicated times, after which Western blotting was used to measure p53 levels. Left panels represent quantified Western blots. (**C, D**) C81 cells were transfected with 280B siRNA or infected with 280B adenovirus and subjected to cell fractionation, followed by Western blotting to measure the cytosolic (C) or nuclear (N) levels of p53 and 280B. Note that the numbers above the lanes represent relative levels of p53 protein, standardized for β-actin levels, with the first lane set to 1. (**E**) C81 cells were infected with empty adenovirus or 280B adenovirus for 48 hrs and then treated with 10 mM Nutlin-3A or Vehicle, followed by Western blotting to measure the cytosolic (C) or nuclear (N) levels of p53 and 280B. For all experiments, β-actin served as a loading control. β-tubulin and RAR, used as markers for cytosolic and nuclear fractions, respectively (C, D, E).

The data above showing that 280B inhibits p53 transcriptional activity and decreases p53 protein levels without changing of its mRNA level suggest that 280B targets the p53 protein. To determine if 280B can affect p53 protein stability, we used cycloheximide (CHX) to measure p53 half-life. Under-expression of 280B by specific siRNA leads to an increase in p53 half-life from 14 min to 24 min (Fig. 4A), while over-expression of 280B decreased weakly the half-life (Fig. 4B).

To better understand the 280B effect on p53 protein stability, we monitored p53 subcellular localization. Note that Western blotting for β-tubulin (cytosolic) and RARα (nuclear) confirmed the purity of the two cell fractions and that the 280B is mainly or exclusively a nuclear protein (Fig. 4C). Reducing endogenous 280B protein levels resulted in significantly elevated nuclear p53 but no change in cytosolic levels (Fig. 4C). The complementary experiment, over-expression of 280B, reduced nuclear p53 without again changing the cytosolic levels (Fig. 4D). These results suggest that 280B promotes p53 proteasomal degradation by inducing p53 nuclear export. To obtain evidence for this, p53 subcellular localization was monitored in the presence of Nutlin 3A, an inhibitor of Mdm2 interaction with p53 and thus nuclear export [31]. As shown in Fig. 4E, Nutlin-3A nearly completely eliminated the 280B-mediated reduction in nuclear p53 levels, a result consistent with a 280B role in p53 nuclear export.

**Figure 5:**
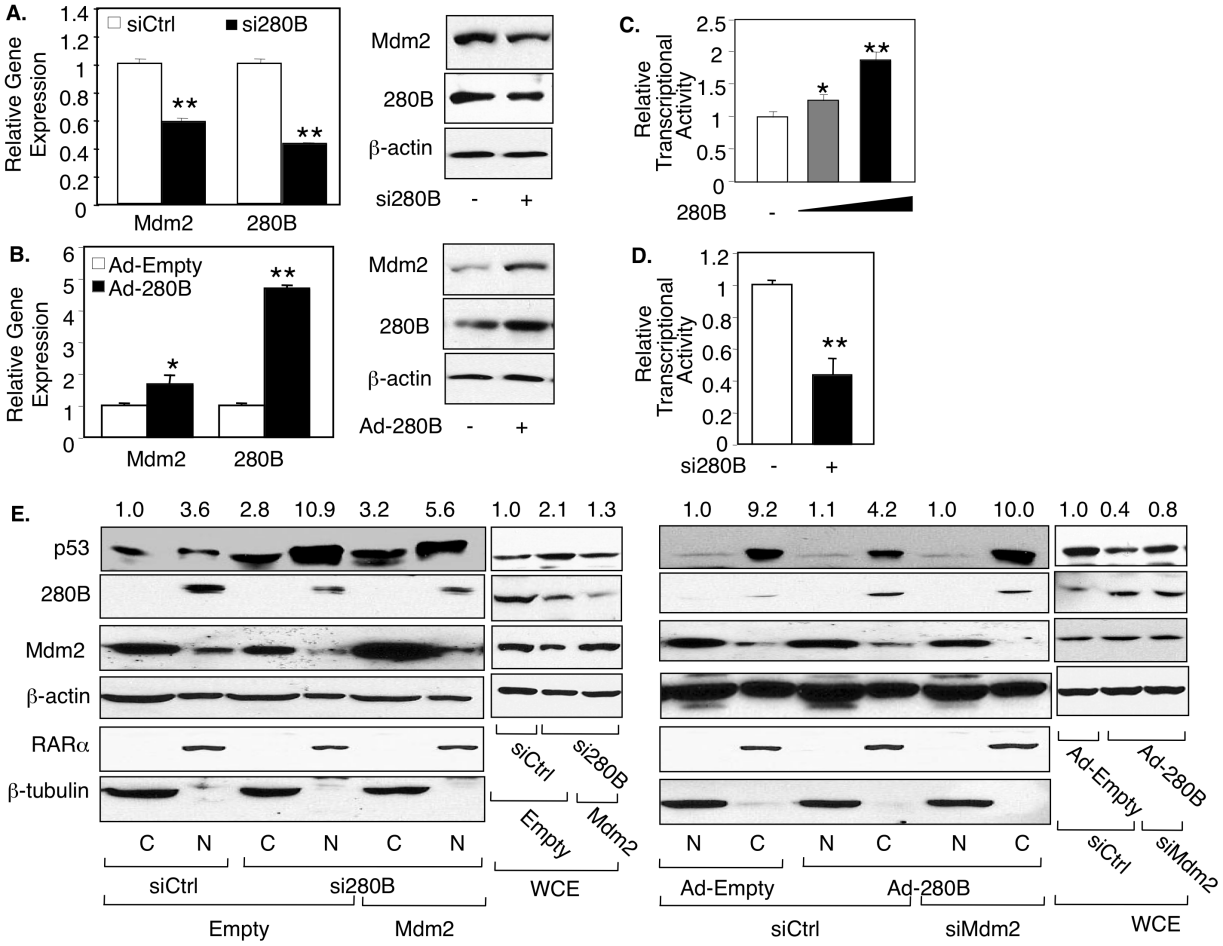
280B down-regulates p53 protein by inducing Mdm2 expression in prostate cancer cells. (**A, B, E**) C81 cells were transfected with (A, E) control siRNA (−) or 280B siRNA (+) or (B, E) infected with Empty adenovirus (−) and 280B adenovirus (+) and subjected to real-time RT-PCR and Western blotting to measure the expression of Mdm2 and 280B. (**C, D**) C81cells were transfected with 0.1 µg Mdm2-Luc and co-transfected with (C) Control siRNA (−) and 280B siRNA (+), (D) Empty pCIneo (−) and 0.2 µg and 0.4 µg 280B, and monitored for 280B activity by measuring Luciferase activity. (**E**) C81cells were co-transfected with empty pCIneo(−) or Mdm2 (+) and control siRNA (−) or Mdm2 siRNA (+), followed by Western blotting to measure the cytosolic (C) or nuclear (N) levels of p53, 280B, and Mdm2. For all experiments, β-actin served as a loading control. β-tubulin and RARa were used as markers for cytosolic and nuclear fractions, respectively (E). All p53 protein levels were relative to the first condition, and it was set to 1. Asterisks * and ** indicate statistical significances of P<0.05 and P<0.01, respectively.

Nuclear export of p53 depends on Mdm2, which is an essential step leading to p53 proteasomal degradation [6], [7]. We confirmed this effect in prostate cancer cells, in which Mdm2 siRNA induced higher levels of nuclear p53 while transient over-expression of Mdm2 reduced these levels (Fig. S4), mimicking the 280 effect on p53. Since ZNF proteins have the ability to regulate transcription, we hypothesized that 280B may affect p53 by regulating the expression of Mdm2. We began to investigate this possibility by measuring the expression of Mdm2, which is significantly reduced at both the mRNA and protein levels when 280B is depleted (Fig. 5A). In contrast, 280B over-expression has the opposite effect, resulting in marked increases in both *Mdm2* mRNA and protein expression (Fig. 5B). Since the *Mdm2* mRNA is affected by 280B, it is possible that the effect is transcriptional. To test this possibility, we analyzed a potential 280B activity on the *Mdm2* promoter, using a luciferase reporter gene assay. Transient expression of 280B in C81 cells increased *Mdm2* promoter activity in a dose-dependent manner (Fig. 5C) and siRNA depletion of 280B blocked *Mdm2* promoter activity (Fig. 5D). These findings clearly demonstrate that 280B acts on the *Mdm2* promoter and suggest that the promoter may be a direct target of 280B.

To begin studying the involvement of Mdm2 in 280B-mediated nuclear export of p53, the Mdm2 expression was altered in C81 cells to determine if this would affect the 280B activity on p53 subcellular localization. Indeed this was the case, as over-expression of Mdm2 significantly reduced the nuclear accumulation of p53 that was triggered by 280B knockdown (Fig. 5E). In the complementary experiment, Mdm2 knockdown eliminated the negative activity that 280B over-expression has on nuclear p53 levels (Fig. 5E). Taken together, these data strongly suggest that the 280B effect on p53 is mediated via 280B regulation of Mdm2 expression. Note that Mdm2 is primarily cytoplasmic in prostate cancer cells.

### 280B is over-expressed in prostate tumors and correlates with reduced p53 protein

**Figure 6:**
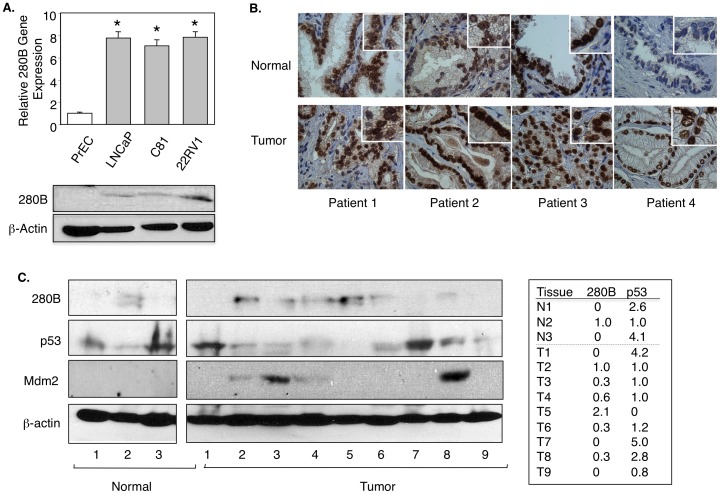
280B is over-expressed in prostate tumors and correlates with reduced p53 protein expression. (**A**)Real-time PCR and Western blotting were used to detect the expression of 280B in normal prostate (PrEC) and prostate cancer cell lines (LNCaP, C81, and CWR-22Rv1). (**B**) Immuno-staining of 280B in 4 matched pairs of prostate tissues, normal versus tumor. (**C**) Cell extract from 4 normal prostate tissues and 9 tumor tissues were subjected to Western blotting to detect 280B, p53, and Mdm2 expression levels. β-actin served as a loading control.

To establish the importance of 280B in prostate cancer, we first examined the expression of 280B in a panel of prostate cancer cell lines and one normal cell line. RT-PCR results showed 280B mRNA levels were 6–7 fold higher in cancer cells than normal prostate epithelial cells (PrEC) (Fig. 6A). Western blotting showed the same finding, with significant protein levels in the prostate cancer cells and nothing detectable in the PrEC cells (Fig. 6A). To extend our observations to the clinical setting, we performed immunohistochemistry on match-paired tissues from 4 patients. Patients 2 and 4 showed stronger 280B immunoreactivity than the normal tissues (Fig. 6B), showing that 280B expression is elevated in some prostate cancer tumors. As would be expected for a transcription factor, 280B is expressed exclusively in the cell nuclei of all tumors (Fig. 6B). The tissue study was expanded to include more tissues and to search for a possible correlation between 280B and p53 expression. As shown in Fig. 6C, 280B protein is expressed in 6 of 10 prostate tumors, while in only 1 of 3 of normal prostate. Interestingly, p53 protein is expressed most highly in those tumors (#1 and #7) that do not express detectable levels of 280B, and the lowest p53 level is found in the tumor with the highest 280B expression. These data display an inverse relationship in expression of 280B and p53 in prostate tumors and thus are consistent with an important role for 280B as a negative regulator of p53 in the prostate cancer.

## Discussion

Prior to this study, 280B was an uncharacterized protein, with the only information available describing its relationship with the large family of zinc finger transcription factors [32]. In this study, we have identified two targets of 280B, sGCα1 and p53, both of which have significant functions in prostate cancer. The first one, sGCα1, has a well-characterized role in NO signaling and a lesser-known role in prostate cancer progression. With respect to the latter role, our previous data have shown that sGCα1 is an important mediator of androgen support of prostate cancer cell proliferation [33] and a repressor of p53 that provides prostate cancer cells a pro-survival function [20]. Our finding that 280B enhances the expression of sGCα1 is consistent with 280B having pro-cancer functions, which are clearly indicated by its high expression in prostate cancer and its pro-proliferative function in prostate cancer cells. Interestingly, the positive effect of 280B was observed on both the sGCα1 mRNA and protein, suggesting that the mRNA, and perhaps the sGCα1 promoter, may be the target of 280B. Using a 1-kb stretch of the sGCα1 promoter on which AR acts [33], we observed no 280B activity (data not shown). This could be due to either the 280B effect on sGCα1 is outside our 1-kb region, or is post-transcriptional, something that will be studied in the future. It is noteworthy that 280B shares a limited common DNA sequence with sGCα1, the sequence that is targeted by siRNA7, which led us to identify 280B. In view of these data, it is intriguing to consider the possibility of a common endogenous miRNA or siRNA that acts on both sGCα1 and 280B. If such a regulatory RNA exists, this RNA would be expected to be down-regulated in prostate cancer since both sGCα1 and 280B are over-expressed in this disease. Identification of such a specific RNA is an important future objective of the lab.

Despite the positive regulation by 280B of sGCα1, over-expression of sGCα1 surprisingly failed to rescue cells inhibited by 280B depletion, suggesting another target of 280B. Our search led to p53. In contrast to sGCα1, p53 is negatively regulated by 280B and this regulation is directed at the p53 protein. Under-expression of p53 is able to almost entirely rescue cells inhibited by diminished 280B expression. Thus, in the context of diminishing 280B expression in prostate cancer cells, which resulted in reduced levels of sGCα1 and increased p53, the increase in p53 was mainly or solely responsible for the reduced cell growth and probably the enhanced apoptosis. This is consistent with previous findings that over-expressed p53 inhibits the proliferation of prostate cancer cells and drives them into apoptosis [34].

Cancer cells use multiple mechanisms to disable the anti-cancer functions of p53 [35]. Mutation of p53 is the most common mechanism, found in over 50% of human cancers [36]. Many proteins have been identified affecting one or more of the other mechanisms [37], including our earlier study demonstrating that sGCα1 can mediate cytoplasmic sequestration of p53 in prostate cancer cells [20]. In this study, we discovered that 280B mediates p53 nuclear export and subsequent protein degradation. To determine how a nuclear 280B, which our data in this report clearly demonstrate, can promote the export of nuclear p53, we looked for a possible 280B-p53 interaction. Immunoprecipitation and immunocytochemistry experiments failed to detect such an interaction (data not shown), leading us to consider another possibility. In view of its nuclear localization and homology to Zinc Finger-containing transcription factors [38], 280B may control the expression of an important regulator of p53 subcellular localization and stability. This regulator turned out to be Mdm2, an important negative regulator of p53 [39]. Our data clearly show that 280B induces expression of Mdm2 by acting on its promoter. This is different from the ZNF protein ZNF668, which stabilizes p53 by preventing Mdm2-mediated p53 ubiquitination and degradation [26] and Apak, which has a negative effect as does 280B but mediated by Apak physical association with p53 [40]. Interestingly, ZNF307 blocks p53 activity by increasing transcription of Mdm2 and EP300 [22]. We do not know at this time about 280B activity on EP300, but our data in this report clearly demonstrate 280B induction of Mdm2 expression. While it was not demonstrated in the previous study [22] that ZNF307 acts on the Mdm2 promoter, the investigators provided data showing that ZNF307 harbors transcriptional activity, making it possible that it may act on the p53 promoter. If it does, then it is possible that ZNF307 and 280B may act together on this promoter. Future work can examine this possibility, as well as the potential that ZNF307, like 280B, is over-expressed in prostate cancer [41]–[44].

The data found here suggest that 280B may act as a transcriptional activator on the Mdm2 promoter. It is also possible that it acts the same way on the sGCα1 promoter, but our cloned 1-kb upstream region of this promoter did not respond to 280B. If 280B does in fact contain transcriptional activation functions, it is difficult to discern that from its amino-acid sequence. What does come from such an analysis is that 280B has 4 C_2_-H_2_ zinc fingers, which represent one of the most common type of DNA-binding domain found in eukaryotic transcription factors [45], thus making it possible that it can act as a transcriptional activator. The analysis also revealed the existence of an N-terminal domain of unknown function found on metazoan proteins that carry a PHD-like zinc finger domain [46]. With respect to this domain, 280B is closely related to the protein UHRF1, which binds to methylated promoters of a number of tumor suppressor genes [47] and can recruit histone acetyltransferases [48] in its role in epigenetic regulation. It is unclear at this time if 280B may have such a role in epigenetic control. As for transcriptional activation domains, no classical domains such as the nuclear receptor AF-2 [49] or the 9-amino acid TAD found in GAL4, p53, and VP16 [50] were identified. Since most activation domains consists of an abundance of amino acids rather than a specific sequence, it is possible that 280B does harbor transcriptional activation activity, similar to the ZNF307 [22], and perhaps a specific type of domain that will not be revealed by amino-acid sequence analysis. Identification of such a domain in 280B will have to wait for truncation analysis using a heterologous DBD, as it has been done for ZNF307 [22].

## Supporting Information

Figure S1 **siRNA7 and siRNA8 have different effects on sGCα1 expression in prostate cancer cells.** (**A, B, C**) LNCaP cells were transfected with control siRNA or two different sGCα1 siRNAs (7, 8) and sGCα1 expression was measured by real-time PCR (A) or Western blotting (B), and cell density was measured by MTT assay (C). Bar graphs represent averages of three independent experiments plus SD. Asterisks indicate statistical significance (P<0.01).
(TIF)

Figure S2 **280B represses p53 activity in prostate cancer cells.** C81 cells were transfected with 0.2 µg p53-Luc and control (−), or 280B expression plasmid (+). Transcriptional activity of p53 activity was quantified by measuring Luciferase activity. All activities are relative to the first condition, and this activity was set to 1. Bar graphs represent averages of three independent experiments plus SD. Asterisks indicate statistical significance (P<0.02).
(TIF)

Figure S3 **Underexpression of 280B induces apoptosis in prostate cancer cells.** LNCaP, CWR22-RV1 cells were transfected with control (−) or 280B siRNA (+), total PARP and cleaved PARP and 280B expression levels were measured by Western blotting.
(TIF)

Figure S4 **MDM2 promotes p53 nuclear export in C81 cells.** C81 cells were transfected with control siRNA or Mdm2 siRNA (**A**) or empty pCIneo or Mdm2 (**B**), and subjected to cell fractionation, followed by Western blotting to measure the cytosolic (C) or nuclear (N) levels of p53 and Mdm2. For all experiments, β-actin served as a loading control. β-tubulin and RAR, used as markers for cytosolic and nuclear fractions, respectively.
(TIF)
