# Supplementary figures and images for: Zinc Finger 280B Regulates sGCα1 and p53 in Prostate Cancer Cells
Source: PLoS One. 2013 Nov 13;8(11):e78766. doi: 10.1371/journal.pone.0078766 (PMC3827277; doi:10.1371/journal.pone.0078766)

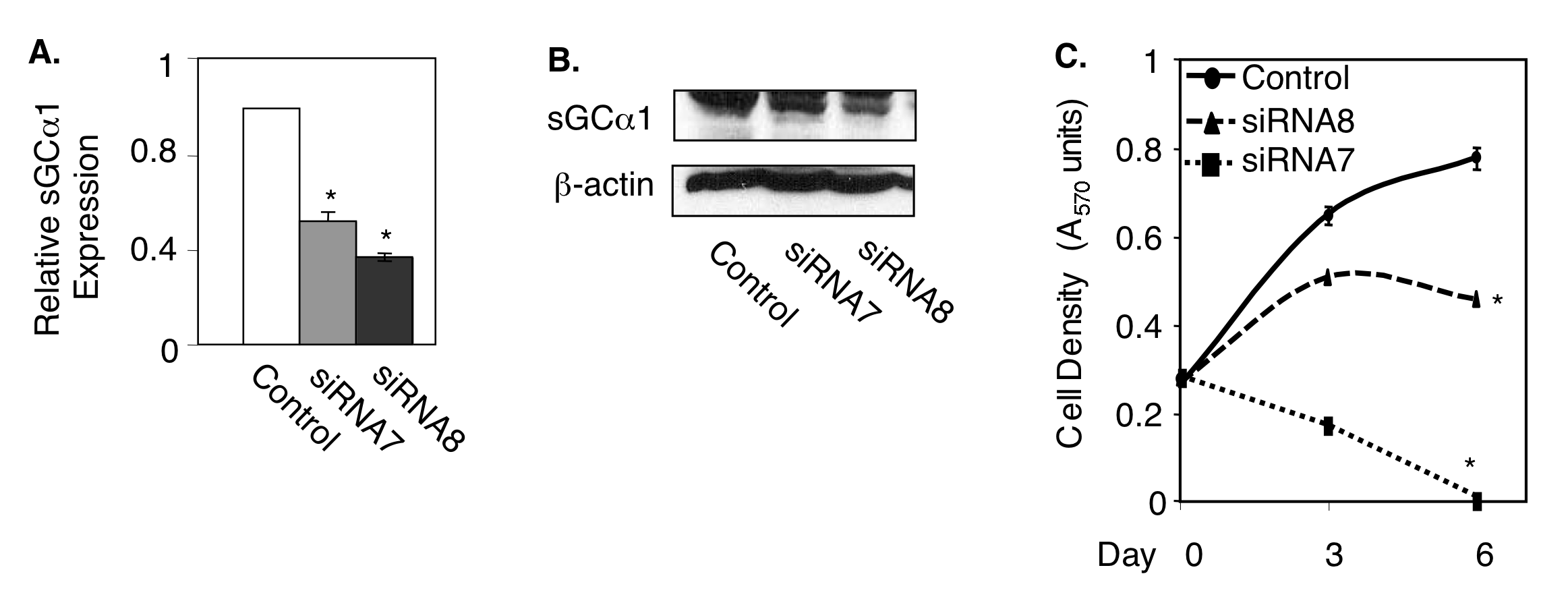

Supplement: Figure S1 — siRNA7 and siRNA8 have different effects on sGCα1 expression in prostate cancer cells. (A, B, C) LNCaP cells were transfected with control siRNA or two different sGCα1 siRNAs (7, 8) and sGCα1 expression was measured by real-time PCR (A) or Western blotting (B), and cell density was measured by MTT assay (C). Bar graphs represent averages of three independent experiments plus SD. Asterisks indicate statistical significance (P<0.01). (TIF) [file pone.0078766.s001.tif]

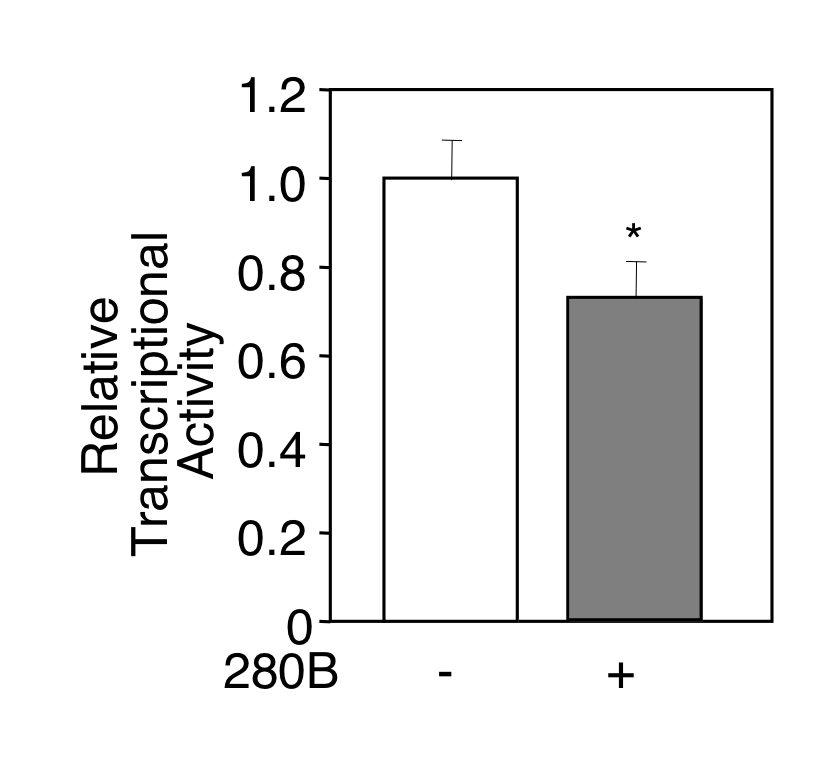

Supplement: Figure S2 — 280B represses p53 activity in prostate cancer cells. C81 cells were transfected with 0.2 µg p53-Luc and control (−), or 280B expression plasmid (+). Transcriptional activity of p53 activity was quantified by measuring Luciferase activity. All activities are relative to the first condition, and this activity was set to 1. Bar graphs represent averages of three independent experiments plus SD. Asterisks indicate statistical significance (P<0.02). (TIF) [file pone.0078766.s002.tif]

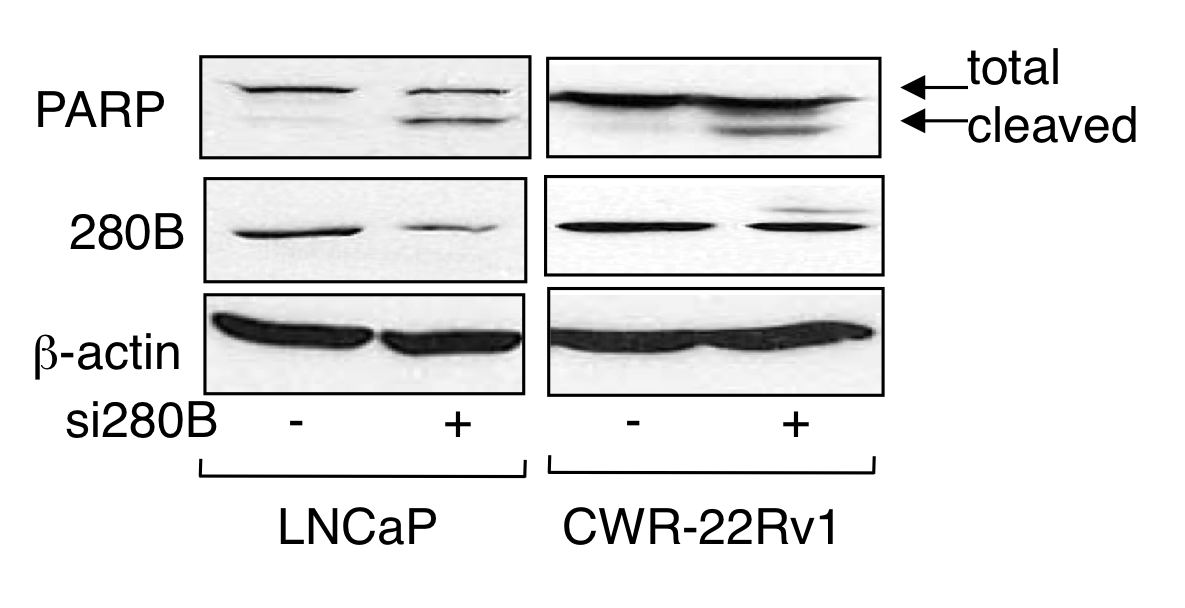

Supplement: Figure S3 — Underexpression of 280B induces apoptosis in prostate cancer cells. LNCaP, CWR22-RV1 cells were transfected with control (−) or 280B siRNA (+), total PARP and cleaved PARP and 280B expression levels were measured by Western blotting. (TIF) [file pone.0078766.s003.tif]

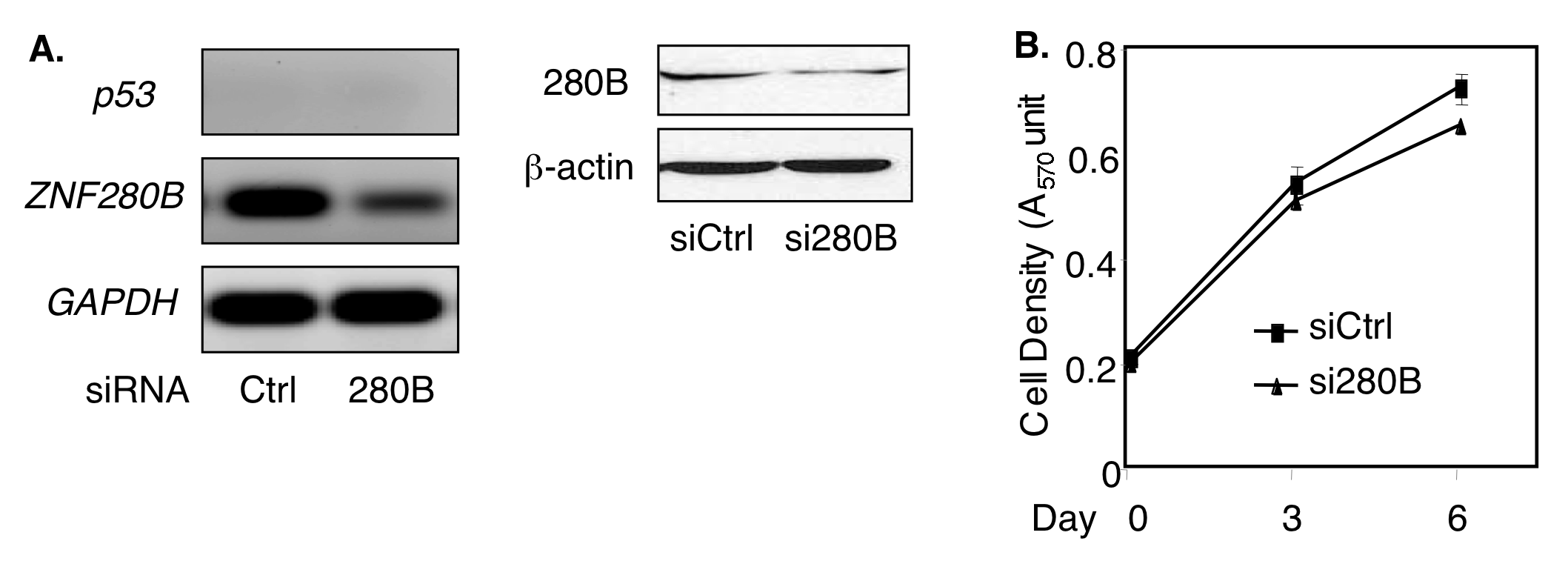

Supplement: Figure S4 — MDM2 promotes p53 nuclear export in C81 cells. C81 cells were transfected with control siRNA or Mdm2 siRNA (A) or empty pCIneo or Mdm2 (B), and subjected to cell fractionation, followed by Western blotting to measure the cytosolic (C) or nuclear (N) levels of p53 and Mdm2. For all experiments, β-actin served as a loading control. β-tubulin and RAR, used as markers for cytosolic and nuclear fractions, respectively. (TIF) [file pone.0078766.s004.tif]
